# The down syndrome biomarker initiative (DSBI) pilot: proof of concept for deep phenotyping of Alzheimer’s disease biomarkers in down syndrome

**DOI:** 10.3389/fnbeh.2015.00239

**Published:** 2015-09-14

**Authors:** Michael S. Rafii, Hannah Wishnek, James B. Brewer, Michael C. Donohue, Seth Ness, William C. Mobley, Paul S. Aisen, Robert A. Rissman

**Affiliations:** ^1^Alzheimer’s Disease Cooperative Study, Department of Neurosciences, UC San Diego School of Medicine, University of California, San DiegoLa Jolla, CA, USA; ^2^Department of Neurology, University of Southern CaliforniaLos Angeles, CA, USA; ^3^Janssen Research and Development LLCTitusville, NJ, USA

**Keywords:** down syndrome, Alzheimer’s disease, biomarkers, amyloid, MRI, PET, plasma, retinal

## Abstract

To gain further knowledge on the preclinical phase of Alzheimer’s disease (AD), we sought to characterize cognitive performance, neuroimaging and plasma-based AD biomarkers in a cohort of non-demented adults with down syndrome (DS). The goal of the down syndrome biomarker Initiative (DSBI) pilot is to test feasibility of this approach for future multicenter studies. We enrolled 12 non-demented participants with DS between the ages of 30–60 years old. Participants underwent extensive cognitive testing, volumetric MRI, amyloid positron emission tomography (PET; 18F-florbetapir), fluorodeoxyglucose (FDG) PET (18F-fluorodeoxyglucose) and retinal amyloid imaging. In addition, plasma beta-amyloid (Aβ) species were measured and Apolipoprotein E (ApoE) genotyping was performed. Results from our multimodal analysis suggest greater hippocampal atrophy with amyloid load. Additionally, we identified an inverse relationship between amyloid load and regional glucose metabolism. Cognitive and functional measures did not correlate with amyloid load in DS but did correlate with regional FDG PET measures. Biomarkers of AD can be readily studied in adults with DS as in other preclinical AD populations. Importantly, all subjects in this feasibility study were able to complete all test procedures. The data indicate that a large, multicenter longitudinal study is feasible to better understand the trajectories of AD biomarkers in this enriched population. This trial is registered with ClinicalTrials.gov, number NCT02141971.

## Introduction

The preclinical/asymptomatic stage of Alzheimer’s disease (AD) has become a target for therapeutic intervention, requiring enriched populations to be more intensively studied. Individuals with Down Syndrome (DS) comprise the largest group with genetically determined AD, with a worldwide population of about six million people. In March 2013, the Alzheimer’s Disease Cooperative Study (ADCS) launched a pilot study named the Down Syndrome Biomarker Initiative (DSBI; Ness et al., 2012). With the DSBI pilot, the ADCS’ goal was to initiate a longitudinal biomarker study similar to the Alzheimer’s Disease Neuroimaging Initiative (ADNI) in individuals with DS, who represent a population highly enriched for developing AD. The ultimate aim of this work is to aid the development of preventive therapies for the dementia associated with both DS and AD, based on the apparent common pathogenic role of beta amyloid (Aβ) in the two conditions.

The tight link between genetic determinants of AD and the overproduction of Aβ provides compelling support for the amyloid cascade hypothesis and has been the focal point in the development of disease-modifying drugs for AD (for review, Sperling et al., 2011). We hypothesize that disease-modifying treatments for AD and DS should begin prior to the onset of cognitive symptoms to prevent extensive neurodegeneration and thus necessitate a clear understanding of biomarker changes throughout the course of the disease.

The study of DS provides a unique opportunity to characterize the preclinical changes associated with predisposition to AD. DS, or trisomy 21, affects 400,000 people in the U.S. with an incidence of 1/691 live births (Parker et al., 2010) and is caused by meiotic non-disjunction, leading to an extra copy of chromosome 21, on which the APP gene resides.

Recent data suggest that AD biomarker changes in DS are similar to those observed in familial and sporadic AD. For example, studies demonstrate a six-fold increase in plasma Aβ in individuals with DS as compared to age-matched non-DS individuals (Schupf et al., 2001, 2007, 2010) and Aβ positron emission tomography (PET) imaging data in DS are consistent with AD patients (Sabbagh et al., 2011, 2015; Handen et al., 2012). Furthermore, as seen in familial and sporadic AD, presence of the Apolipoprotein E (ApoE) ε4 allele is generally associated with greater accumulation of Aβ plaques in the brains of adults with DS (Hyman et al., 1995; Lemere et al., 1996). Presence of ApoE ε4 allele is also associated with an earlier age of onset of dementia (Schupf et al., 1996; Deb et al., 2000; Coppus et al., 2008; Prasher et al., 2008).

Postmortem studies indicate that adults with DS have a similar, prominent pattern of cerebral atrophy involving the medial temporal lobe structures, as seen in the early stages of AD (Hof et al., 1995; Teipel et al., 2004; Mullins et al., 2013). Volumetric magnetic resonance imaging (MRI) studies of age-related brain changes in DS demonstrate the same pattern of hippocampal-specific atrophy observed in AD. Furthermore, the hippocampal atrophy in DS correlates with changes in memory measures (Krasuski et al., 2002; Beacher et al., 2010). Hypometabolism on regional fluorodeoxyglucose (FDG) PET also correlates with onset of dementia in older adults with DS (Schapiro et al., 1992a,b; Pietrini et al., 1997).

In this study, we collected structural MRI, Aβ PET, FDG PET, retinal Aβ, plasma Aβ species, and cognitive performance measurements in a cohort of 12 non-demented adults with DS aged 30–60. Our goal was to establish feasibility of conducting a biomarker-intensive study in adults with DS.

## Materials and Methods

### Study Design and Participants

The DSBI pilot enrolled 12 non-demented subjects for a 3-year longitudinal study of AD biomarkers (see Table 1 for Schedule of Events). The present analysis is restricted to the baseline data. Four non-demented subjects were in each age range: 30–40, 40–50 and 50–60. Inclusion criteria limited enrollment to individuals having a chromosome karyotype of DS due to Trisomy 21. Subjects were required to have a caregiver, absence of other neurological and psychiatric disorders, and be capable of and willing to perform study procedures. Having a clinical diagnosis of dementia was considered exclusionary as was presence of 6 months of progressive cognitive or functional decline as per ICD-10 criteria (Sheehan et al., 2015). Exclusion of a diagnosis of dementia was also based on absence of evidence of recent deterioration in cognitive function found not secondary to medical illness (e.g., hypothyroidism, sleep apnea) in conjunction with absence of a significant decline in function over a period of 6 months or more. The diagnosing neurologist was experienced with dementia in DS and incorporated diagnostic recommendations from the National Task Group on Intellectual Disabilities and Dementia Practices (Moran et al., 2013). All participants or their legal representatives provided written informed consent before partaking in the study in accordance with the regulations and approval of the ethics committee at the University of California, San Diego, La Jolla, CA, USA.

**Table 1:** **Schedule of events for DSBI pilot**.

Visit	Screen/BL	YR1	YR2	YR3 (Comp)
Month	0	12	24	36
**Study Procedures**				
*Screening/administrative*
Informed consent [/assent]	x			
Inclusion/exclusion criteria	x			
Medical history and demographics	x
*Safety assessments*
Physical examination	x	x	x	x
Vital signs	x	x	x	x
*Neurocog assessments*
Scales, questionnaires, etc.	x	x	x	x
*Clinical* *laboratory assessments*
Hematology, Chemistry	x	x	x	x
Urinalysis	x	x	x	x
Pharmacogenomics (DNA)
ApoE	x
*Biomarkers (eg, plasma, serum sample collection)*
Plasma, serum collection	x	x	x	x
*Imaging*
Tau PET			x	x
Amyvid PET	x			x
FDG PET	x			x
vMRI	x	x	x	x
Retinal amyloid imaging	x	x	x	x
*Ongoing subject review*
Concomitant therapy	x	x	x	x
Adverse events	x	x	x	x

### Procedures

Between March 2013 and January 2014, we collected data from participants including plasma samples, neuropsychological evaluations, neurological examination, ApoE genotyping, volumetric MRI, amyloid PET, FDG PET, retinal Aβ imaging, and clinical assessment. Subjects came for five visits over a 5-week period for assessments to be made. Events occured in the following order: visit 1: neuropsychological and clinical assessment, neurological examination; visit 2: Amyloid PET; visit 3: MRI and blood draw; visit 4: FDG PET, visit 5: retinal Aβ imaging.

#### Cognitive, Behavioral, and Functional Assessments

Cambridge Neuropsychological Test Automated Battery (CANTAB). The CANTAB was used to assess cognition. The CANTAB is a computerized touch-screen assessment of neuropsychological function composed of a number of tests (Luciana, 2003; Smith et al., 2013). The tests selected from this battery for this study were as follows: motor control (MOT): the subject is asked upon appearance of a crossmark on the screen, to touch it as quickly and accurately as possible using the index finger of their dominant hand. This is essentially a practice routine to become skilled with regards to touchscreen use. The outcome parameter is median reaction time (RT): the subject is asked to hold the index finger on the holding button on the button box and keep it pressed until a circle on the screen lights up and then touch that circle with the index finger as quickly and accurately as possible. In the Simple condition, there is only one possible circle that will light up (Simple RT). In the five-choice condition, any of five circles can light up (five-Choice RT). Paired associated learning (PAL): the subject is shown 2–8 (max) distinct visual patterns, each at one of eight positions inside of an octagon on the screen. The task is to memorize which pattern occurred where. After the memorization stage, each pattern is shown in the center of the screen and the subject has then to touch one of eight possible positions where the pattern first occurred.

Repeatable Battery for the Assessment of Neuropsychological Status (RBANS) was developed for the dual purposes of identifying and characterizing abnormal cognitive decline in the older adult and as a neuropsychological screening battery for younger patients (Randolph et al., 1998). It is a brief, individually administered test that can be used to measure cognitive decline or improvement. The full battery is composed of 12 subtests assessing the: immediate memory, visuospatial abilities, language, attention and delayed memory. In this study, seven subtests of the RBANS were used to assess immediate and delayed memory, as well as the language capacities (subtests: list learning, story memory, list recognition, list recall, picture naming, semantic fluency, digit span).

Vineland-II Adaptive Behavior Scale (VABS-II) parent/caregiver interview form. The VABS-II measures personal and social skills such as communication, daily leaving skills, and socialization and will provide a composite score reflecting an individual’s overall function. In addition, the optional maladaptive behavior index could be used. The survey interview form was administered to parents or caregivers using a semi-structured interview format (Sparrow and Havis, 2005).

Observer Memory Questionnaire-Parent Form (OMQ-PF). The OMQ-PF is a 27-item questionnaire designed to ascertain parents’ perceptions of the subject’s memory function. This questionnaire is comprised of items inquiring about memory function in everyday scenarios (Gonzalez et al., 2008).

Anxiety Depression and Mood Scale (ADAMS). The ADAMS is a well validated, 28 item behavior-based informant instrument designed to be used specifically with individuals with developmental disabilities to assess anxiety, depression and mood disorders (Esbensen et al., 2003). Points given for each behavior the caregiver endorses. Subscales (5) include: Manic/Hyperactive, Depressed Mood, Social Avoidance, General Anxiety, Compulsive Behavior. The ADAMS possesses a satisfactorily high alpha, with a mean alpha of 0.80 in each of the 28 items. The mean item test-retest correlation is 0.789.

Cambridge Examination for Mental Disorders of Older People with Down’s Syndrome and Others with Intellectual Disabilities (CAMDEX-DS). Cognitive status was measured using the Cambridge Cognitive Examination (CAMCOG), the cognitive section of CAMDEX, a composite index of episodic memory, orientation, language, attention, praxis and executive function previously validated for use in DS (Hon et al., 1999). The CAMCOG is appropriate for assessing cognitive function in people with intellectual disability, unlike more standard tests of cognitive function such as the Wechsler Adult Intelligence Scales. The CAMCOG incorporates, and is highly correlated with, the Mini Mental State Examination (MMSE; Blessed et al., 1991).

Dalton Dyspraxia scale for Adults with DS: evaluates simple sequences of voluntary movements expected to deteriorate with the onset and progression of dementia in AD among persons at all levels of premorbid intellectual disability. Participants are given points for each task they are able to perform (Dalton, 1992).

The Goodenough–Harris Draw-A-Person Test: brief paper and pencil mental age test. This assessment system analyses 14 different aspects of a drawing done by the subject (such as specific body parts and clothing) for various criteria, including presence or absence, detail, and proportion. In all, there are 64 scoring items. A standard score is recorded for the drawing, and a mental age is assigned based on this score (Goodenough and Harris, 1950).

### Biofluid Collection

Blood, (separated into plasma and serum), was collected to accommodate the assay of the broadest range of the best antecedent biomarkers/analytes. Blood samples were drawn in two lavender-capped EDTA tubes and one red-capped BD tube. One lavender-capped tube was centrifuged at 3000 rpm for 10 min to separate plasma for storage. Ten milliliter of the plasma sample was aliquoted into barcoded polypropylene vial and frozen at −80. The second blood tube was used for serum extraction, which will be processed by allowing the samples to clot at room temperature, spun as above for plasma preparation, aliquoted and stored in barcoded polypropylene tubes at −80. The third blood tube was used for DNA isolation using Qiamp DNA blood maxi kit (Qiagen). All biosamples were processed and stored at the ADCS Biomarker Core using standard operating procedure.

### Plasma Aβ Analysis and Internal Standard

Banked plasma was assayed, quantified, and quality controlled by the ADCS Biomarker Core using the MesoScale Validated Aβ triplex (Aβ 38, 40, 42) according to the manufacturer instructions. Each assay plate also included an internal standard which provided a means for adjusting plate-to-plate variation and assessing freezer storage effects, as previously described (Donohue et al., 2014). To mitigate plate-to-plate variability, plates were purchased in bulk and run consecutively.

### Real Time PCR for Apolipoprotein E (ApoE) Genotyping

Genotyping for ApoE alleles was performed using real time PCR Restriction Fragment Length Polymorphism analysis by the ADCS Biomarker Core according to standard operating procedures. ApoE genotyping was performed using Applied Biosystems TaqMan SNP Genotyping Assay (C_3084793_20 and C_904973_10 corresponding to ApoE SNPs rs429358 and rs7412, respectively). The assay was run on a Bio-Rad CFX96 Touch Real Time PCR Detection System, using a cycling program of 98 C for 2 min. and 39 cycles of 98 C for 15 s and 62 C for 45 s five positive controls for each genotype and one negative control were included in each plate to ensure accurate determination.

### Neuroimaging

#### Volumetric MRI

The MRI protocol included series to assess for structural pathology (T2-weighted fluid attenuated inversion recovery, T2*-weighted gradient recalled echo, and diffusion weighted imaging) along with a series modeled on the non-accelerated T1-weighted sequence from ADNI for volumetric processing (3D inversion recovery prepared spoiled gradient recalled imaging; inversion time 500, flip angle 10, 1.25 mm × 1.25 mm in-plane resolution, 156 sagittal slices with 1.2 mm spacing). Scanning was performed on a 1.5 Tesla GE Signa HDxt scanner, and radiologist overread was performed on all scans to identify any clinically significant incidental findings. NeuroQuant image preprocessing and automated segmentation was used to measure brain structure volumes (Brewer et al., 2009; Kovacevic et al., 2009; Heister et al., 2011). Briefly, this includes corrections for gradient non-linearities (Jovicich et al., 2006) and intensity non-uniformity (Sled et al., 1998) and application of probabilistic-atlas-based segmentation to automate measurement of multiple brain regions (Fischl et al., 2002). The procedure is cleared by the U.S. Food and Drug Administration and the European Medicines Agency for use in automating the identifying, labeling, and quantifying the volume of segmental brain structures identified on MR images (21 CFR 892.2050). To minimize multiple comparisons, for analysis, a single measure of medial temporal atrophy that comprises hippocampal volume loss and temporal horn ex-vacuo dilatation, “Hippocampal occupancy (HOC),” was calculated as described previously (Heister et al., 2011). This measure is simply H/(H + T), where H is hippocampal volume and T is temporal horn volume.

#### FDG PET

FDG PET procedures were based on those used in ADNI.^1^ Subjects were asked to fast for at least 6 h prior to the scanning session. Subjects’ blood glucose was checked prior to scanning and was required to be <180 mg/dL. After the injection of 5 mCi of 18F-FDG, subjects were kept in a quiet, dimly lit room with eyes and ears unoccluded for 30 min, after which they were placed in the Siemens EXACT HR+ 961 PET tomograph (CTI, Knoxville, TN, USA), which yielded 63 transverse sections spaced 2.43 mm apart with a 15.5 cm field of view (FOV) in 3D mode and 5 mm in-plane spatial resolution full width at half maximum (FWHM). Images were acquired at an angle parallel to the cantho-meatal plane and reconstructed using a ramp filter (cut-off frequency = 0.5 cycles/pixel) into 128 × 128 pixel images. Each subject was placed in a headholder during scanning to allow accurate positioning using a low-power neon laser. Data were acquired as 6 × 5 min frames, followed by a positron transmission scan. Frames were averaged and all images were coregistered to the individual’s native space MRI. For signal normalization, the brainstem was used as a reference region.

#### Florbetapir F 18 PET

Subjects received IV injections of 10 mCi of Florbetapir F 18 and after 40 min of uptake, 10 min of emission data were collected by the Siemens EXACT HR+ 961 PET tomograph (CTI, Knoxville, TN, USA), which yielded 63 transverse sections spaced 2.43, 3.5 mm apart with a 15.5 cm FOV in 3D mode, with 4 mm in-plane spatial resolution (FWHM). Images were acquired at an angle parallel to the cantho-meatal plane and reconstructed using a Hann filter (cut-off frequency = 0.5 cycles/pixel) into 128 × 128 pixel images. Each subject was placed in a headholder during scanning to allow accurate positioning using a low-power neon laser. All PET scans were supervised. Statistical analysis was performed as for FDG, except, for florbetapir, the cerebellum was used as the reference region for signal normalization.

#### Retinal Aβ

The NeuroVision Retina HD is a fundus camera that is substantially equivalent to the FDA approved cameras currently utilized in clinical practice. In this procedure, a filter set matched to the fluorescence characteristics of curcumin is utilized for retinal amyloid plaque imaging *in vivo*. Quantitative analysis of Aβ plaque number, area (μm^2^) and distribution are performed from retinal images. For the acquisition, the same exposure settings and the same gain values are used for all images. The emission signals of Aβ plaques stained with curcumin are compared to the background signals in the retinal tissue, to determine signal-to-background ratio.

At the visit, subjects had auto-fluorescence imaging and curcumin fluorescence imaging of the right retina. Patients were asked to take a standard over the counter oral vitamin E supplement for each retinal amyloid imaging visit, beginning at Day 1 and continuing through day 3 of imaging. Patients were dosed with curcumin twice daily for 2 days; At Day 1, patients commenced taking oral curcumin. On Day 2, subjects had another day of ingesting curcumin. On Day 3, subjects had auto-fluorescence imaging, and curcumin fluorescence imaging. NeuroVision calculated the retinal amyloid index in a blinded fashion for each subject.

#### Statistical Analysis

For cognitive, imaging analyses and fluid biomarker assessment, ApoE4 carriers and non-carriers were compared in terms of their age, educational level, clinical ratings, and neuropsychological test scores using Wilcoxon and Pearson’s Chi-square tests. We also estimated Spearman rank correlations for each selected pairs of continuous measures. These correlation analyses were group by variable type: (1) cognitive vs. imaging; (2) cognitive vs. retinal and plasma; and (3) MRI vs. PET. We controlled the false discovery rate within each of these groups (Benjamini and Hochberg, 1995). This pilot study is not well powered. All analyses should be considered exploratory and any findings need to be confirmed with larger sample sizes. The sample size of *n* = 12 provides approximately 80% power to detect only correlations larger than *ρ* = 0.84 with two-sided FDR *α* = 5% and assuming 90% of null hypotheses are true. Similarly, *n* = 6 subjects per ApoEε4 group provides approximately 80% power to detect only large standardized group differences of  *δ* = 2.66.

## Results

**Figure 1:**
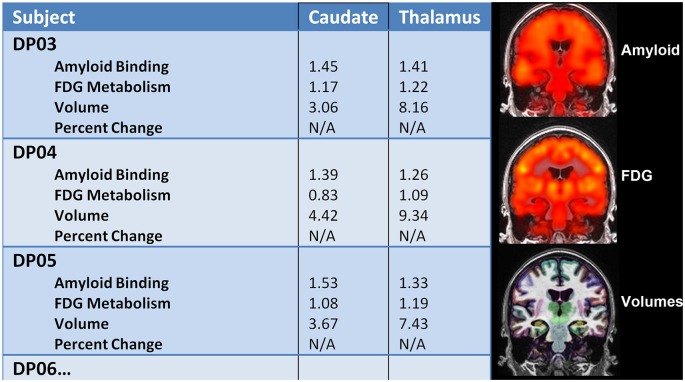
**Left:** multimodal comparisons can be made in native space within individual subjects longitudinally. **Right:** Amyloid PET, FDG PET, and volumetric MRI were successfully performed in adults with down syndrome (DS) to capture important structure-function relationships.

All 12 participants completed all required testing. Tables 2 and 3 show demographic characteristics for the 12 non-demented participants included in this study, grouped by APoE4. Of the 12 subjects, 50% (*n* = 6) were ApoE4 carriers. All of the ApoEε4 non-carriers were female, while four of the ApoEε4 carriers were female. These two groups did not differ significantly in demographics, clinical ratings, or neuropsychological test scores. All subjects were amyloid positive, but to varying degrees. Table 4 provides a line listing of some of the key neuroimaging variables for each study participant sorted by posterior cingulate gyrus (PCG) amyloid PET. Figure 1 demonstrates how multimodal assessments are made across subjects in native space.

**Table 2:** **Participant characteristics in DSBI feasibility study**.

	N (E4−)	N (E4+)	Total (*N*)
*ApoE*	6	6	12
Gender: F	6	4	10
Gender: M	1	1	2
Age	43.5 (9.8)	47.2 (7.4)	45.0 (9.8)
**Educ. years:**
0	0	1	1
12	2	5	7
18	4	0	4

*There were six subjects who were ApoE4 positive and six who were ApoE4 negative. Average age was 45 (S.D. 9.8)*.

**Table 3:** **Cognitive and functional performance summaries by ApoE4 geneotype**.

	N	E4−	E4+	Combined	*P*-value
**Direct testing**					
CAMCOG-DS	12	58.2 (23.4)	56.5 (20.3)	57.3 (20.9)	0.81
Goodenough-DAP	12	14.83 (9.47)	17.33 (2.73)	16.08 (6.78)	0.81
RBANS Composite	12	259.3 (30.1)	249.7 (47.1)	254.5 (38.0)	0.47
RBANS Digit span	12	4.67 (2.07)	3.33 (2.66)	4.00 (2.37)	0.27
RBANS List recall	12	14.67 (3.98)	11.83 (3.54)	13.25 (3.89)	0.18
RBANS Memory	12	50.00 (6.66)	49.50 (13.17)	49.75 (9.96)	0.68
RBANS Language	12	70.8 (19.9)	60.7 (16.3)	65.8 (18.2)	0.22
Delayed Memory	12	43.00 (3.79)	43.17 (7.76)	43.08 (5.82)	0.44
CANTAB-Total	12	87.5 (33.2)	109.0 (33.6)	98.2 (33.8)	0.29
Dalton Dyspraxia	12	205.7 (41.3)	177.7 (49.9)	191.7 (46.1)	0.31
**Informant-based**					
ADAMS	12	17.8 (17.4)	17.8 (23.3)	17.8 (19.6)	0.81
Vineland-2	12	124.217 (32.9)	96.767 (24.5)	110.4 (31.2)	0.24
OMQ-PF	12	91.5 (27.1)	89.8 (27.3)	90.7 (26.0)	0.69

*Higher score on all cognitive tests indicates better performance. The only exception is higher score on ADAMS, which indicates increased symptoms of anxiety, depression, agitation. ADAMS, Anxiety Depression and Mood Scale; CAMCOG-DS, Cambridge Examination for Mental Disorders of Older People with Down’s Syndrome and Others with Intellectual Disabilities-Cognitive scale; Goodenough DAP, Goodenough-Harris Draw a Person test; RBANS, Repeatable Battery for the Assessment of Neuropsychological Status; (CANTAB), Cambridge Neuropsychological Test Automated Battery; OMQ-PF, Observer Memory Questionnaire (Parent Form)*.

**Table 4:** **Amyloid PET and FDG PET with hippocampal volume and retinal amyloid index**.

Subject	Age	Mental age	ApoE 4	Amyloid PET clinical read	Grey matter Amyloid PET (SUVr)	FDG PET clinical read	Average Hippocampal volume (cm^3^)	Average Hippocampal Occupancy (%)	Retinal amyloid index	
DP06	37	9	E3-E3	Negative	0.938	Normal	3.52	73	1.63
DP01	32	7	E3-E3	Negative	0.97	Mildly hypo	3.12	78	2
DP07	34	7	E2-E4	Negative	0.988	Normal	3.37	75	2.47
DP08	39	5	E3-E3	Positive	1.054	Hypo	3.19	82	1.8
DP02	45	3	E2-E3	Positive	1.171	Hypo	2.99	45	2.2
DP12	45	6	E3-E4	Positive	1.176	Hypo	2.91	75	1.83
DP05	48	8	E3-E3	Positive	1.177	Hypo	3.47	77	1.68
DP11	47	7	E3-E4	Positive	1.245	Hypo	3.48	71	2.34
DP13	50	8	E3-E4	Positive	1.344	Hypo	3.14	60	1.58
DP04	55	6	E3-E4	Positive	1.385	Hypo	3.01	45	1.7
DP03	52	7	E3-E4	Positive	1.401	Hypo	3.25	51	2.2
DP09	60	7	E3-E3	Positive	1.457	Hypo	2.73	60	—

*Areas with higher amyloid deposition have relative hypometabolism on FDG PET. The listing is sorted with respect to increasing grey matter amyloid PET signal*.

### Correlation Analyses

**Figure 2:**
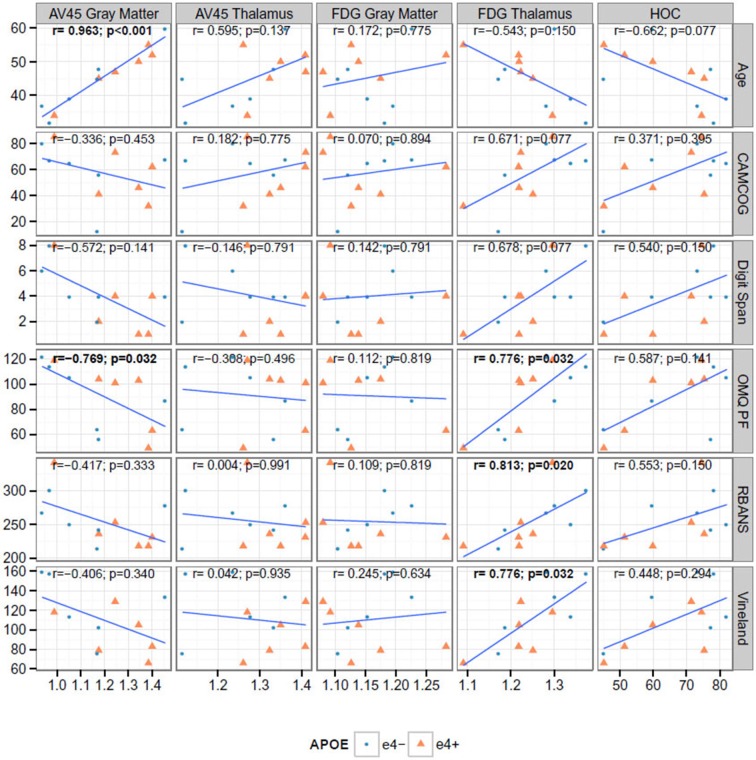
**Correlations between cognitive and neuroimaging measures**. The bold text indicates Spearman rank correlations (*r*) that are significant at the 0.05 level after false discovery rate adjustment. AV45 = ^18^F-florbetapir.

Figure 2 shows the correlation between key neuroimaging, and age and cognition. Age was significantly associated with florbetapir (AV45) uptake in the gray matter (*r* = 0.963, *p* < 0.001) and thalamus (*r* = 0.595, *p* < 0.041), and HOC (*r* = −0.662, *p* = 0.019). Florbetapir uptake in the gray matter was also significantly correlated with OMQ PF (*r* = −0.769, *p* = 0.005032). FDG uptake in the thalamus was significantly correlated with CAMCOG, Digit Span, OMQ PF, RBANS, and Vineland (all *r* > 0.6 and *p* < 0.01). HOC was correlated with OMQ PF (*r* = 0.587, *p* = 0.049). Only the correlations between FDG Thalamus and OMQ-PF; FDG Thalamus and Vineland; and Florbetapir (AV45) Gray Matter and age were significant at the 0.005 level (*r* = 0.776, *r* = 0.776, and *r* = 0.963 respectively).

### Correlation Between Cognition, and Plasma Aβ and Retinal Amyloid

**Figure 3:**
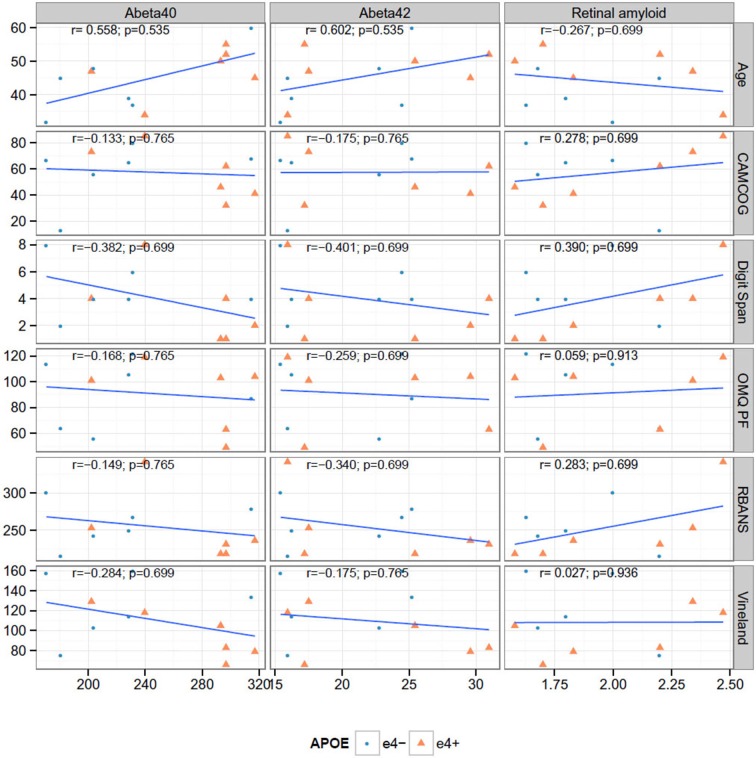
**Correlations between cognitive measures, retinal amyloid and plasma biomarkers**. None of the Spearman rank correlations (*r*) are significant at the 0.05 level after false discovery rate adjustment.

**Figure 4:**
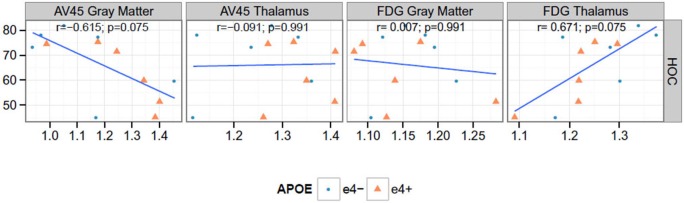
**Correlations between PET and hippocampal occupancy (HOC)**. None of the Spearman rank correlations (*r*) are significant at the 0.05 level after false discovery rate adjustment. AV45 = ^18^F-florbetapir.

Figure 3 shows the correlation between key neuroimaging, and age and cognition. We found no significant correlations between plasma or retinal amyloid measures and with age or cognition (Figure 3); nor between MRI and PET (Figure 4). We did find a significant correlation between A beta 42 and age (*r* = 0.602, *p* = 0.038).

### Correlation Between PET measures and HOC

We found a significant negative correlation between Florbetapir (AV45) uptake in gray matter and HOC (*r* = −0.615, *p* = 0.037). FDG uptake in thalamus and HOC were positively correlated (*r* = 0.671, *p* = 0.020) see Figure 4.

### Retinal Amyloid Imaging

**Figure 5:**
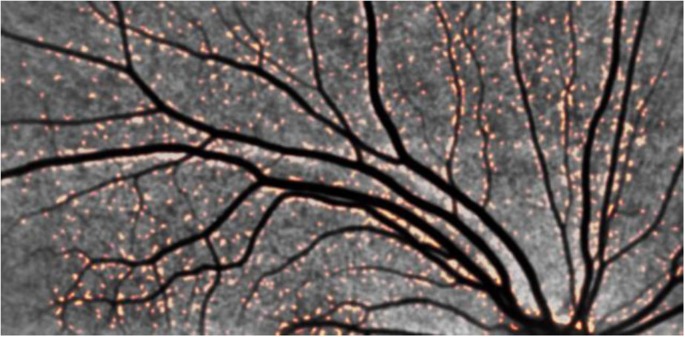
**Representative retinal images from an adult with DS demonstrating positive amyloid plaques in DS**. Note the orange-colored puncta. The distribution in the vicinity of blood vessels is striking, pointing to a retinal manifestation of congophilic angiopathy.

We imaged amyloid plaques in the retina of all subjects in this small cohort, Figure 5. All subjects demonstrated amyloid positivity.

## Discussion

Although limited in sample size, this small pilot study provides strong support for the feasibility of a multicenter longitudinal AD biomarker study in adults with DS. Our findings also show that prior to dementia onset, changes in volumetric MRI, amyloid PET and FDG PET and plasma are detectable and consistent with preclinical AD in adults with DS. Adults with DS had elevated levels of plasma Aβ1–42 concentrations and plasma Aβ1–42:Aβ1–40 ratios. These findings are consistent with previously published findings for individuals with DS (Schupf et al., 2010). Consistent with previous autopsy studies, most subjects demonstrated amyloid PET positivity reflecting fibrillar amyloid plaque deposition.

We also find adults with DS can tolerate amyloid-β deposition without significant effects on cognitive functioning. This has been reported by others (Hartley et al., 2014) and likely reperesents the preclinical stage of AD.

### Study Strengths

We successfully studied AD biomarkers in all participants with DS, who, in the absence of an effective prevention treatment, are certain to develop symptoms of AD. With this cohort, we confirm feasibility of a large-scale multicenter longitudinal study designed to characterize trajectories of cognitive decline. The fact that DS has native wild-type APP may make it more relevant to studying biomarkers applicable to the general sporadic AD. Additionally, we compared several different brain imaging and fluid biomarker measurements, as well as exploratory biomarkers such as retinal Aβ imaging, to characterize some of the earliest biomarker changes associated with the predisposition to AD.

### Limitations and Issues of Interpretation

This study also has several limitations, including small sample size, absence of longitudinal data, and uncertainty in the extent to which our findings are generalizable to other causes of late-onset AD. Although the retinal amyloid findings should be regarded as exploratory, the uncorrected significance levels, bilateral pattern, and resemblance to the pattern reported previously in patients with AD reduce the likelihood that they are attributable to the type I error associated with multiple regional comparisons. Although our findings are currently limited to DSBI pilot participants, we have sought to harmonize our biomarker measurements and undertake biological fluid assays in the same laboratory used by investigators in the study of other DS cohorts (LonDowns and Fundació Catalana de Síndrome de Down), thus providing complementary data and converging evidence in the preclinical study of AD in DS patients.

Additional studies are needed to clarify several issues: the extent to which the structural and functional abnormalities identified in young adults with DS at genetic risk for AD precede Aβ plaque deposition; whether these changes are neurodegenerative or developmental; whether or not there is any cerebral fibrillar Aβ deposition in young adults with DS.

### Conclusion

Adults with DS have volumetric MRI, Aβ PET, FDG PET and retinal Aβ changes, along with plasma biomarker findings consistent with Aβ1–42 overproduction. This study shows some of the earliest known AD biomarker changes in adults with DS and underscores the need for studies to clarify the earliest brain changes associated with the predisposition to AD. We have recently added Tau PET imaging to the set of biomarkers assessed in this cohort. Under the auspices of the DSBI pilot, we are continuing to characterize the age-related trajectory of biomarker changes associated with preclinical AD to set the stage for the first clinical trial of an anti-Aβ therapy in the preclinical treatment of AD in adults with DS.

## Author Contributions

MR, PSA, SN, RR and WM designed the study. MR, HW, JB, MD and RR executed the study, performed the research, analyzed the data and wrote the manuscript.

## Conflict of Interest Statement

The primary sponsor of the study, Janssen, provided input on study design as well as review of this manuscript. All authors had full access to all the data in the study and had final responsibility for the decision to submit for publication. Seth Ness is an employee of Janssen.

## Footnotes

^1^http://www.adni-info.org/Scientists/doc/ADNI2_PET%20Tech_Manual-Version_4_2014Oct27_CLEAN.pdf
